# The health disparities cancer collaborative: a case study of practice registry measurement in a quality improvement collaborative

**DOI:** 10.1186/1748-5908-5-42

**Published:** 2010-06-04

**Authors:** David A Haggstrom, Steven B Clauser, Stephen H Taplin

**Affiliations:** 1 VA Health Services Research & Development Center on Implementing Evidence-based Practice, Roudebush VAMC, Indianapolis, IN, USA; 2 Division of General Internal Medicine and Geriatrics, Department of Medicine, IU School of Medicine, Indianapolis, IN, USA; 3 Indiana University (IU) Center for Health Services and Outcomes Research, Regenstrief Institute, Inc., Indianapolis, IN, USA; 4 Division of Cancer Control and Population Sciences, National Cancer Institute, Bethesda, MD, USA

## Abstract

**Background:**

Practice registry measurement provides a foundation for quality improvement, but experiences in practice are not widely reported. One setting where practice registry measurement has been implemented is the Health Resources and Services Administration's Health Disparities Cancer Collaborative (HDCC).

**Methods:**

Using practice registry data from 16 community health centers participating in the HDCC, we determined the completeness of data for screening, follow-up, and treatment measures. We determined the size of the change in cancer care processes that an aggregation of practices has adequate power to detect. We modeled different ways of presenting before/after changes in cancer screening, including count and proportion data at both the individual health center and aggregate collaborative level.

**Results:**

All participating health centers reported data for cancer screening, but less than a third reported data regarding timely follow-up. For individual cancers, the aggregate HDCC had adequate power to detect a 2 to 3% change in cancer screening, but only had the power to detect a change of 40% or more in the initiation of treatment. Almost every health center (98%) improved cancer screening based upon count data, while fewer (77%) improved cancer screening based upon proportion data. The aggregate collaborative appeared to increase breast, cervical, and colorectal cancer screening rates by 12%, 15%, and 4%, respectively (p < 0.001 for all before/after comparisons). In subgroup analyses, significant changes were detectable among individual health centers less than one-half of the time because of small numbers of events.

**Conclusions:**

The aggregate HDCC registries had both adequate reporting rates and power to detect significant changes in cancer screening, but not follow-up care. Different measures provided different answers about improvements in cancer screening; more definitive evaluation would require validation of the registries. Limits to the implementation and interpretation of practice registry measurement in the HDCC highlight challenges and opportunities for local and aggregate quality improvement activities.

## Background

Concerns about the quality of healthcare delivery have increased in recent years, reflecting data that suggests a lack of adherence to evidence-based practice [1,2]. Cancer care has not been immune to these concerns as research has demonstrated gaps in quality throughout the cancer care continuum [3]. In response, healthcare organizations have attempted to close these gaps by developing interventions for quality improvement. Some third-party payers have developed indirect incentives for quality improvement by reimbursing providers using pay-for-performance metrics [4], and pay-for-performance demonstration programs sponsored by Medicare have addressed cancer screening [5]. Fundamental to quality improvement and pay-for-performance are valid measures of quality or performance, but small practices may be limited by the small number of events relevant to any single disease and the burden of data collection [6]. Little has been reported about the implementation challenges of measurement in smaller practice settings. The Health Disparities Cancer Collaborative (HDCC) [7] provides an example of quality improvement incorporating practice registry measurement among community health centers.

The HDCC emphasizes plan/do/study/act (PDSA) cycles [8] that identify deficiencies in quality, deliver interventions, and measure the resulting change. Rapid PDSA cycles leverage multiple, small practice-level interventions that are refined and increased in scale to improve processes of care. The HDCC builds upon the Breakthrough Series (BTS) collaborative model, in which approximately 20 health centers are brought together in an organized manner to share their experiences with practice-level interventions, guided by practice-based measurement. In this manuscript, we use the HDCC as a case study for the implementation of practice registry measurement in a multi-center quality improvement collaborative.

In the US, approximately one-half of physician organizations have any disease registry; furthermore, one-half of these registries are not linked to clinical data [9]. The HDCC encouraged practice registries to track patient populations eligible for cancer screening and follow up, commonly independent of an electronic medical record. Previous evaluations of collaborative activity have used self-reported practice registry data [10], enhanced practice registry data [11], or bypassed practice registry data in favor of chart audit [12].

However, direct knowledge from practice about the implementation of practice registries, and interpretation of the data collected, is rare in the medical literature [6,13]. This paper addresses several key measurement issues worth consideration by stakeholders participating in any quality improvement intervention: How complete are the data across health centers over time? For what types of care processes is it feasible to detect changes in care? And what answers do different approaches to presenting practice change provide? The answers to these questions provide insights into explanations for data reporting patterns, as well as how practice registry measurement can be interpreted at different levels. This information may guide quality improvement for cancer screening and follow up, and assist local and national decision-makers in using practice registry data collected for other clinical practices or problems.

## Methods

### Setting

Sixteen community health centers, supported by the Health Resources and Services Administration (HRSA), participated in the HDCC. HRSA directs its resources toward financially, functionally, and culturally vulnerable populations [14]. Basic characteristics of the 16 health centers participating in the HDCC are described in Table 1. The collaborative activities were led and supported by HRSA, the Centers for Disease Control and Prevention, and the National Cancer Institute (NCI).

**Table 1:** Health center characteristics

Patients eligible for screening at health center level*	Mean (range)
Breast	849 (86 to 3305)

Cervical	1,556 (131 to 5,195)

Colorectal	549 (82 to 3466)

Number of months reporting any registry data*	17 (12 to 18)

Number of providers (physicians, nurse practitioners, physician assistants)**	52 (7 to 205)

Number of nurses (registered nurses, licensed practical nurses)**	34 (1 to 103)

Region of health centers***	Number (proportion)

Northeast	3 (19%)

Midwest	4 (25%)

South	7 (44%)

West	2 (13%)

*obtained from practice registry software

**obtained from survey of health center financial officers

***per U.S. census region categories

### Collaborative intervention

From 2003 to 2004, the HRSA HDCC administered the BTS, a collaborative model [15] developed by the Institute for Healthcare Improvement (IHI) [16]. The HDCC adapted elements from the 'chronic care model' to improve the quality of cancer screening and follow up. The chronic care model is defined by six elements: healthcare organization, community linkages, self-management support, decision support, delivery system redesign, and clinical information systems [17]. The HDCC's learning model involved three national, in-person sessions and the expectation that local teams would be organized at health centers to pursue PDSA cycles relevant to cancer screening. The 16 centers were selected through an active process that involved telephone interviews with health center leaders to assess their enthusiasm and willingness to commit the resources necessary for success. The local teams consisted of employees with multiple backgrounds and roles, including providers (physicians, physician assistants, and nurse practitioners), nurses, appointment staff, and laboratory and information systems personnel. The effort and staff time allocated averaged four full-time equivalent (FTE) per team with an aggregate of 950 hours per team. Participating health centers reported performance measures to each other and central facilitators, and talked by teleconference monthly.

### Performance measures

HDCC measures of screening and follow up for breast, cervical, and colorectal cancer were collected over 15 months in the collaborative (See Additional File 1 for full description of the performance measures). These measures assessed four critical steps in the cancer care process: the proportion of eligible patients screened, the proportion screened receiving notification of results in a timely manner, the proportion of abnormal results evaluated in a timely manner, and the proportion of cancer cases treated in a timely manner [18]. Screening measures were based upon United States Preventive Services Task Force (USPSTF) guidelines and finalized through a process of discussion and group consensus among collaborating health centers. These performance measures were similar to the cancer screening measures developed by the National Committee for Quality Assurance (NCQA) [19] and the Physician Consortium for Performance Improvement, sponsored by the American Medical Association (AMA) [20]. In contrast to other measurement systems, the HDCC did not exclude age-appropriate individuals due to medical reasons or patient refusal (as was done by the Physician Consortium for Performance Improvement). Conversely, other systems did not incorporate timely follow-up (notification, evaluation, or treatment) as part of their indicator sets.

### Practice registry data collection

Health centers reported the size of the patient population who were eligible for screening and follow up and received screening and follow up every month from September 2003 through November 2004. Information was reported to HDCC facilitators from HRSA, NCI, and IHI. We obtained Institutional Review Board approval, as well as written consent from each participating health center, to use the self-reported practice registry data.

Community health centers each created a practice registry of individuals eligible for screening or follow up among patients who had been seen in the health center at least one time in the past three years. All health centers participating in the HDCC used the practice registry data software provided by the HDCC; nationwide, HRSA community health centers were encouraged, but not mandated, to use the software. Data entry varied from the wholesale transfer of demographic information from billing data queried for age-appropriate groups to hand entry.

In 2000, HRSA supported the development and deployment of electronic registry software. Over the next five years, HRSA continued to support numerous iterations of the registry software to address both the increasing scope of the collaboratives (such as cancer screening) and the needs of clinicians and other frontline-staff users. Informing this process was an advisory group of health center clinicians and technical experts that provided insight and guidance about critical registry functionalities and the needs of measurement to effectively support practice management. Training in the software was provided by HRSA at a national level, as an adjunct to collaborative learning sessions, and at the regional and local level by the Information System Specialist (ISS). The training typically consisted of four- to eight-hour interactive sessions in which participants would have a 'live' experience on laptops.

The registry software assembled individual patients seen at the health center into an aggregate population to share with other HDCC sites. The data were posted on a secure data repository to be shared with HDCC facilitators and benchmarked against other health centers. A data manager from the medical records department at each center who had training in use of the registry uploaded the data.

The process of entering patients into the practice registry fell into two general categories: a process whereby patients seen at the center in the previous month were entered into the practice registry as they were seen, and a process whereby patients who had been seen at the center before the previous month were entered into the practice registry based on the criterion of being seen at least once in the past three years. The number of patients described as eligible in any given month represented the number of patients that the health center had so far been able to enter into the practice registry. Eligible patients in the practice registry were then searched on the last work day of each month to identify who had received screening or follow up within an appropriate timeframe. The number of patients who were up-to-date with screening or follow up was reported and shared among collaborative participants on a monthly basis; no shared information was identifiable at the patient level.

### Analyses

We anticipated a start-up period of about three months when the practice registry would be in the process of being implemented at the health centers. To test this assumption, we determined the completeness of monthly registry data reported by each health center over the first three months (September 2003 through November 2003) and the last 12 months (December 2003 through November 2004). Within each interval, we determined the proportion of months when data were not reported from each health center (center-months). Preliminary analyses confirmed our initial assumptions: during the first three months of the collaborative, 12.5% of the months over which reporting was possible were absent for screening mammography. For screening Pap test, 10.4% of months were absent; and for colorectal cancer screening, 16.7% were absent. This level of missing data was more than twice as high as was observed during the last 12 months of data reporting (see Results); and consequently, we chose to focus subsequent analyses on the last 12 months of the collaborative. Analyses were performed across 16 health centers over 12 months, thus, data reporting was possible for a total of 192 center-months.

We conducted three primary analyses:

1. To determine the completeness of practice registry data for screening and follow up across health centers over time, we described the proportion of health centers who reported or had data available for at least two points in time (months) for each cancer care process (Table 2).

**Table 2:** Health centers reporting practice registry data in ≥ two months for each cancer care process

	Number of health centers reporting	Percentage of health centers reporting
**Cancer Screening**		

Women with mammogram in last two years (age ≥42 years)	16	100.0%

Women with pap test within last three years (age ≥21)	16	100.0%

Adults appropriately screened for colorectal cancer (age ≥51)	16	100.0%

**Breast cancer follow-up and treatment**		

Women notified of mammogram results within 30 days	8	50.0%

Women with follow-up evaluation of abnormal mammogram completed within 60 days	2	12.5%

Women with breast cancer starting treatment within 90 days	1	6.25%

**Cervical cancer follow-up and treatment**		

Women notified of Pap test results within 30 days	10	62.5%

Women requiring colposcopy completing evaluation within 90 days	3	18.75%

Women with CIN 2,3 starting treatment within 90 days	2	12.5%

**Colorectal cancer follow-up and treatment**		

Adults notified of colorectal cancer screening results within 30 days	6	37.5%

Adults with follow-up evaluation of positive FOBT within 8 weeks	5	31.25%

Adults with colon polyps or cancer starting treatment within 90 days	2	12.5%

2. To determine for which cancer care processes it would be feasible to detect differences in the proportion of patients who received care, we calculated the detectable change statistic for each process (Table 3). For example, if 20% of patients received screening, we determined what additional proportion of patients would have to receive screening, given the same sample size, to be significantly different from 20%. For the two-sided tests, our assumptions were that the threshold for detecting differences was 5% (alpha = 0.05) and the power was 80% (beta = 20%). These calculations were performed using the power procedure from SAS 9.1 [21]. Based upon power and completeness, we chose to focus subsequent analyses on only cancer screening, not timely follow-up or treatment.

**Table 3:** Populations receiving and eligible for cancer care processes at beginning of evaluation period for aggregate collaborative

Cancer care process	Eligible population	Process received	Eligible	Detectable change*
**Cancer screening**				

Mammography	Women age ≥42	2,373	10,522	2%

Pap test	Women age ≥21	8,446	20,114	2%

Colorectal cancer screening	Adults age ≥51	1,855	7,760	3%

**Breast cancer follow-up and treatment**				

Documented notification of mammogram results within 30 days	Women receiving mammogram	674	2,373	6%

Additional evaluation within 60 days of abnormal mammogram	Women with abnormal mammogram	30	125	24%

Initial treatment within 90 days of diagnosis	Women diagnosed with breast cancer	2	31	44%

**Cervical cancer follow-up and treatment**				

Documented notification of Pap test results within 30 days	Women receiving Pap test	2,325	8,446	3%

Colposcopy evaluation within three months of abnormal Pap test	Women requiring colposcopy based on Pap test	73	292	15%

Initial treatment within 90 days of diagnosis	Women diagnosed with CIN2,3	8	34	47%

**Colorectal cancer follow-up and treatment**				

Documented notification of colorectal cancer screening results within 30 days	Adults receiving colorectal screening	575	1,855	6%

Colonoscopy (or sigmoidoscopy and BE) within eight weeks of positive testing	Adults with abnormal FOBT	29	123	24%

Initial treatment within 90 days of diagnosis	Adults diagnosed with colon polyps or cancer	1	33	40%

*80% power to detect this amount of change at significance level of 0.05 (two-sided)

3. To describe and test practice change in the health centers, we used two main approaches: for the aggregate collaborative, we performed a chi-squared test comparing the proportion of individuals screened at the beginning and end of the collaborative evaluation period; and for each individual health center, we conducted the same before/after comparison and then determined the proportion of individual chi-squared tests that were significant among all health centers.

4. To generate trend figures for individual health centers, we charted the number and proportion of individuals who were screened--as well as the number eligible--for breast, cervical, and colorectal cancer at the beginning (December 2003) and end (November 2004) of the collaborative evaluation period. The three screening tests had nine potential combinations--or patterns of change--among the number of individuals screened, the number of individuals eligible, and the proportion of individuals screened.

## Results

### Practice registry data reporting patterns

During the 12-month period under evaluation, self-reported practice registry data were available from 16 community health centers for screening mammography in 95%, or 182/192 of the center-months over which reporting was possible. For screening Pap test, data were available for 95% of the center-months, and for colorectal cancer screening, data were available for 94% of the center-months.

All participating health centers reported practice registry data regarding cancer screening (Table 2). The proportion of health centers who reported practice registry data for other care processes were the following across different cancers: documented notification of screening test results (37 to 63%); evaluation of abnormal screening test results (12 to 32%); and delivery of treatment within an adequate time frame after cancer diagnosis (6 to 13%).

### Detectable change

The HDCC as a whole had large enough numbers of women and men eligible for screening mammography, screening Pap test, and colorectal cancer screening to detect a change of 2% to 3% in cancer screening (Table 3). Likewise, the numbers of individuals who received breast, cervical, and colorectal cancer screening tests were large enough to detect a 3% to 6% change in the documented notification of each screening test result within 30 days. The numbers eligible were such that only a 15% to 24% change could be detected in the additional evaluation of abnormal screening test results, and only a change of 40% or more could be detected in the delivery of treatment within an adequate time frame after cancer diagnosis.

### Different approaches to presenting practice change

#### Individual versus aggregate level

For the aggregate HDCC, the proportion screened at the beginning and end of the evaluation period increased for breast, cervical, and colorectal cancer by 12%, 15%, and 4%, respectively (p < 0.001 for all comparisons, Table 4). For individual health centers, the before/after chi-squared test of proportions demonstrated a statistically significant change in screening among less than one-half of health centers (Table 4).

**Table 4:** Before/after comparisons at aggregate collaborative and individual health center level

		Cancer screening		
		**Women with mammogram in last two years (age ≥42 years)**	**Women with pap test within last three years (age ≥21)**	**Adults appropriately screened for colorectal cancer (age ≥51)**

**Aggregate collaborative**	Before numerator	2,373	8,446	1,855
	
	Before denominator	10,522	20,114	7,760

	After numerator	4,508	13,898	3,307

	After denominator	13,003	24,300	11,968

	Before proportions	23%	42%	24%

	After proportions	35%	57%	28%

	Before/after chi-squared test	p < 0.001	p < 0.001	p < 0.001

Individual health centers (out of 16 possible health centers)	Increase in before/after counts	15/16 (94%)	16/16 (100%)	16/16 (100%)
	
	Increase in before/after proportions	12/16 (75%)	11/16 (69%)	14/16 (88%)

	Before/after chi-squared test significant	7/16 (44%)	6/16 (38%)	5/16 (31%)

#### Counts versus proportions

Across breast, cervical, and colorectal cancer, almost all health centers had an increase in the number screened (98%, 47/48). The denominator here (48) is composed of each screening test (three tests) measured at each health center (16 centers). Most health centers (88%, 42/48) also had an increase in the number eligible for cancer screening. Fewer health centers (77%, 37/48) had an increase in the proportion of individuals screened.

**Figure 1:**
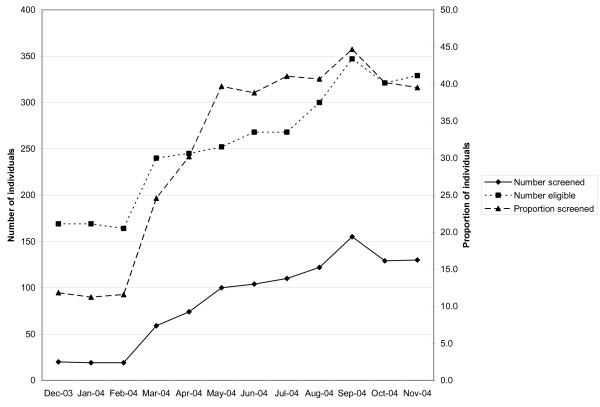
**Individual health center wherein number of individuals screened for breast cancer increased, number eligible increased, and proportion screened increased**.

**Figure 2:**
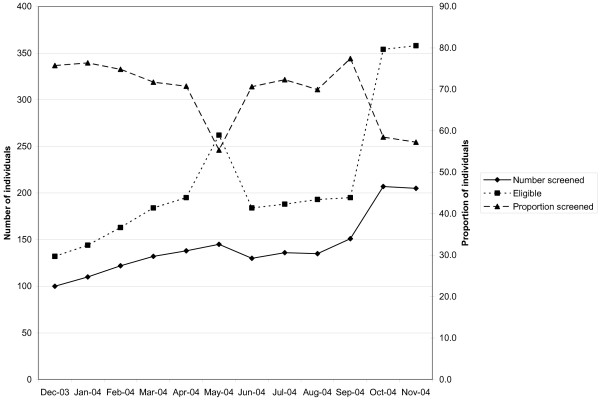
**Individual health center wherein number of individuals screened for breast cancer increased, number eligible increased, and proportion screened decreased**.

**Figure 3:**
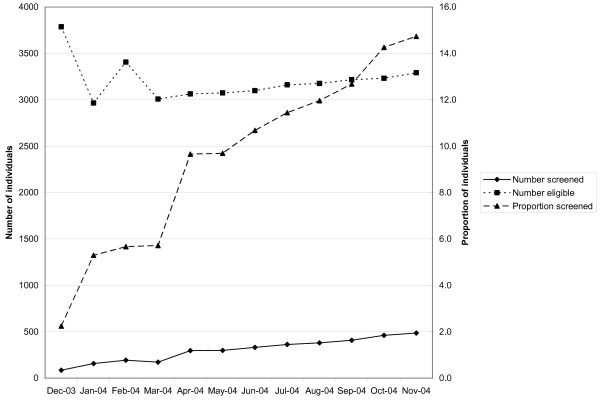
**Individual health center wherein number of individuals screened for breast cancer increased, number eligible decreased, and proportion screened increased**.

Among health centers participating in the collaborative, three different combinations--or patterns of change--emerged across the following measures: the number of individuals screened, the number of individuals eligible, and the proportion of individuals screened. Table 5 provides complete data across the sixteen reporting health centers. The three patterns (described in Figures 1, 2 and 3 using representative breast cancer screening examples from an individual health center) were as follows: the majority of the time (65%, or 31/48), the number screened, the number eligible, and the proportion screened all increased (Figure 1); occasionally (23%, 11/48), both the number screened and number eligible increased, while the proportion screened decreased (Figure 2); and less often (13%, 6/48), the number screened increased, while the number eligible decreased. Logically, the proportion screened increased in each instance (Figure 3). At the individual health center level, patterns of change tended to track together across the three types of screening. At two centers, the second pattern of change (Figure 2) occurred across breast, cervical, and colorectal cancer screening, and at another center, across breast and cervical cancer screening. At two centers, the third pattern of change (Figure 3) occurred across both breast and cervical cancer screening.

**Table 5:** Changes from baseline to final measurement in the number of individuals screened, the number eligible, and the proportion screened across cancer screening tests

	Mammography screening	Pap test screening	Colorectal cancer screening
	**Screened/Eligible/Proportion**	**Screened/Eligible/Proportion**	**Screened/Eligible/Proportion**

CHC 1	13/**-1**/15.6	16/**-9**/14.9	20/3/16.6

CHC 2	37/72/28.9	69/113/**-9.8**	31/66/30.7*

CHC 3	105/226/**-18.5**	135/323/-25.6*	46/224/**-11.8**

CHC 4	513/347/24.2*	807/996/3.9	298/214/19.3*

CHC 5	78/258/6.2	746/817/57.0*	58/158/20.4*

CHC 6	110/160/27.7*	427/444/37.5*	28/135/3.9

CHC 7	60/**-84**/4.3*	1133/710/23.9*	290/58/16.7*

CHC 8	205/252/14.1	296/341/12.0	140/153/7.9

CHC 9	351/730/**-3.9**	972/1379/**-3.9**	299/536/**-7.7**

CHC 10	69/114/10.0	125/153/23.0	34/109/2.8

CHC 11	400/**-497**/12.5*	759/**-2552**/24.8*	151/1747/2.6*

CHC 12	215/328/8.7*	220/453/5.2	86/416/0.1

CHC 13	6/51/**-2.1**	41/90/0.5	51/74/0.9

CHC 14	133/166/14.5*	270/404/7.9	86/146/6.3

CHC 15	27/184/2.0	10/219/**-1.5**	29/146/3.3

CHC 16	1/251/-18.4*	183/422/**-4.7**	6/**-21**/2.8

CHC: community health center; bold italics indicate a decrease in the number or proportion of individuals screened or eligible

*p < 0.05

## Discussion

There were challenges in this evaluation that raise issues relevant to measuring and improving practice. The challenge of collaborative measurement begins with the question of the completeness of the practice registry data and how they were collected, as well as the nature of the performance measures and the populations involved. In the HDCC, both practice registry data completeness and the feasibility of detecting change varied by cancer care process. For cancer screening, every health center reported data, and data were reported for most months. Furthermore, enough individuals were eligible for cancer screening so that relatively small improvements were detectable. On the other hand, because additional evaluation of abnormal tests or timely initiation of treatment were reported infrequently, only relatively large changes were detectable.

Practice registry data from HDCC community health centers can be interpreted and guide action on at least two levels: the individual health center and the aggregate collaborative. Aggregate measures suggested improvement in the HDCC as a whole across all cancer screening processes (breast, cervical, and colorectal); however, individual health center screening measures captured improvement among a minority of health centers. Individual health centers acting alone may not have adequate statistical power for traditional research purposes, but nonetheless, collecting their own practice registry data can enable practice directors, providers, and staff to function as learning organizations [22] to understand their own data, as well as share their local understanding with other health centers participating in the same type of quality improvement activities. At the aggregate level, practice registry data shared among multiple health centers may inform other large collaborative or quality improvement efforts, as well as policymakers, akin to a multi-site clinical trial.

### Explanations for practice registry data reporting patterns

As the HDCC progressed to healthcare processes more distal to the initial screening event, the number of health centers reporting practice registry data decreased, and the size of the detectable change increased. In the HDCC, reporting practice registry data on the follow up of abnormal results and treatment of cancer was voluntary. Both the small number of events reported, and centers that reported them, commonly made it infeasible to test for statistically significant changes in follow up or treatment, even over the entire collaborative. The small number of abnormal screening results reported--and the even smaller number of cancer diagnoses--have at least three primary explanations: the frequency of these care processes or events was indeed small; the medical information was available in a local medical record but the health centers did not report these events in automated form to the HDCC program, even when they did occur; and health centers did not have routine access to the medical information necessary to report the measures because the care occurs outside their practice.

### Frequency of different care processes

At any single health center, it is possible that no cancers were detected during the period of time under evaluation (about 3 in 1,000 screening mammograms detect a breast cancer [23]), but it seems very unlikely that any given health center would not have any abnormal results to report (approximately 1 in 10 screening mammograms are abnormal [24]). Because all health centers were not reporting all data describing each cancer care process, selection bias clearly threatens the validity of general inferences drawn from the data collected in the overall collaborative.

### Why information may be available locally, but not reported to the HDCC

As demonstrated by example in the case of the HDCC, a larger number of eligible patients allows more precise measurement of practice performance [6]. A primary care population usually has enough individuals eligible for cancer screening so that multiple health centers--joined together by a collaborative--have sufficient power to detect small changes in screening. Of the screening follow-up steps reviewed, the highest percentage of health centers reported timely notification of Pap test results (62.5%), most likely because these services were performed onsite at the health centers. Yet overall, the same level of precision and power possible for screening was not possible for the measures and comparisons of diagnostic follow-up or treatment events. Therefore, health centers in the HDCC may have felt less accountable for reporting care processes that occurred infrequently knowing the limitations of measuring these clinical processes [25].

Health centers may have had concerns about how misascertainment of only a few cases could potentially make their overall performance appear much worse. Concerns about negative perceptions have allegedly driven reporting behavior in other settings. For example, health maintenance organizations were more likely to withdraw from voluntary Healthcare Effectiveness Data and Information Set (HEDIS) measure disclosure when their quality performance was low [26]. Reinforced by concerns about the potential negative perceptions of their employees or other health centers, participating health centers may have chosen not to invest their limited time and resources into reporting voluntary measures with few events.

### Why health centers may not have access to the data necessary to report the measures

The limited ability of the HDCC to detect changes in additional evaluation or treatment also was a function of the clinical setting in which HDCC measurement took place--community health centers delivering primary care. Compared to the number of abnormal tests identified in a primary care practice, more abnormal tests will be found in procedural settings (*e.g*., mammography centers and gastroenterology practices) where these tests are performed across multiple, referring primary care practices. Similarly, more cancer diagnoses will be found where cancer patients are treated (*e.g*., oncology and surgery practices) from multiple, referring practices.

On a practical level, primary care health centers may simply not have routine access to the medical record data necessary to report information related to diagnostic follow-up and treatment. There was no uniform workflow for data from institutions outside the HDCC, and we suspect that the lack of access to data outside the primary care practices contributes most to the small number of abnormal screening results and cancer diagnoses reported. Health policy experts have emphasized that single-practice data systems are insufficient for effective care coordination across practices [27]. For example, the extremely low rate of timely treatment after cancer diagnoses among reporting health centers (3 to 24%) very likely represented the lack of a systematic way to collect feedback from oncology practices rather than quality gaps; data across practices is very difficult to locate outside the context of integrated data and delivery systems. Health centers appeared to report what little information was available regarding follow-up and treatment and shift their focus to cancer screening. In the subsequent HDCC regional collaborative, substantial emphasis was placed upon building communities of practice to help address the lack of coordination between primary care and subspecialty practices [28]. Community health centers may perceive it as unfair to hold primary care practices accountable for whether or not their referral was evaluated or treated in a timely fashion given that the clinical delivery (and financial benefit) of these services falls within the scope of other practices in the healthcare system. In the HDCC, this perception may have further contributed to non-reporting of such distal events, even though on a system level, appropriate and timely follow-up is essential for a successful cancer screening program.

In assigning accountability for performance, one general approach is that any individual provider is held accountable for those activities directly under his/her control. This approach is taken in measurement systems supported by the AMA's Physician Consortium for Performance Improvement for physician office practices. An alternative approach to assigning accountability would be the integration of performance measurement across multiple healthcare settings to capture services across the full continuum of cancer care. Here the accountability is placed upon the healthcare system--or a network of physicians--as opposed to individual providers. Building upon our HDCC experience, policymakers may want to consider new methods to identify and reward the team of providers responsible for the care of patients with complex medical conditions [29,30], including cancer. Policymakers may also want to consider our findings as reinforcing evidence of the need for patient-centered medical homes [31], if they make additional resources available for coordinating care with other providers and using data systems to track referrals and results.

### Practice registry data interpretation

#### Individual level

Over the course of the collaborative, health centers consistently increased the absolute number of individuals screened, yet on occasion, both the number of individuals eligible for screening and the proportion screened declined. Figures 1, 2 and 3 provide examples of the three patterns observed at the health center level during the course of the HDCC. The interpretation of these various patterns may be helpful to both collaborative group leaders and individual practices trying to understand their own data.

Two main interpretations are possible when the number screened increases. Either more screening is occurring at the health center, or the same amount of screening is occurring at the health center but more complete measurement of screening is occurring. Two parallel interpretations exist when the number eligible for screening changes. Either the eligible population is changing, or the eligible population is stable but a different proportion of the eligible population is being identified or measured.

Informal observations gathered from individuals involved with the self-report of practice registry data provide some insight into likely explanations for these patterns. HDCC participants suggested that health centers struggled to establish a reliable denominator population eligible for screening. The early, sharp drop in the number eligible in Figure 3 is likely attributable to the establishment of a more reliable denominator population in the first several months of the HDCC, rather than a sudden drop in the eligible population. Similarly, the sharp rise and drop in the number eligible midway through the HDCC in Figure 2 likely represents a mid-course correction in how the eligible, denominator population was ascertained. Finally, the late, rapid increase in the eligible population in Figure 2 likely represents the inclusion of a bolus of patients by new automated data collection, as opposed to rapid growth in the eligible population served by the health center.

The observation that unique patterns of change tracked across different cancer screening tests at the same center further suggests that explanations related to data collection and entry most likely drive these patterns. Using registries to track screening is a new organizational process for many practices [9]. These centers received training, but training does not replace actual practice experience in allowing organizations to become proficient. Practices are likely to encounter problems at first, and thus, there may be considerable imprecision in the first year of data.

When the danger of an unreliable eligible population denominator exists, tracking the numerator (number screened) may be the best way to chart progress, provided the numerator itself is reliable. Challenges in establishing the denominator population are not unique to the HDCC, and, in fact, the HDCC likely represented a best-case scenario of particularly motivated health centers with special attention from national organizations. A minority of clinical practices has any disease registry to provide guidance in managing the care of their patients [9]. Furthermore, cancer screening typically involves many more patients than any other specific disease (for example, diabetes) because screening takes place among healthy populations defined largely by age thresholds. Ultimately, a paradigm shift to population-based information systems and healthcare delivery may be necessary to track and manage the delivery of clinical preventive or screening services.

The experience of the HDCC suggests that the data entry burden for large screening populations poses significant challenges for primary care practices [6], as well as regional or national policymakers interested in organizing such practices in larger quality improvement efforts. Formal assessment of the burden of data entry and tracking activities upon health center personnel would inform estimates of the cost of other collaboratives targeting large populations. Sudden trend shifts trigger questions about the quality of the practice registry data when they occur. Although some centers performed automated data transfers from billing systems to registries, this process required advanced data management capabilities that were not always available [32,33]. Complete registries will be difficult to implement until community health centers are equipped with a full electronic medical record system, accompanied by functionalities designed to manage the health of populations.

The nature and intensity of practice registry measurement may appropriately change for different purposes. For example, quality improvement programs like the HDCC need to focus most upon threats to internal validity when performing before/after assessment within a single, or collective group, of health centers. On the other hand, pay-for-performance activities typically reward practices differentially based upon their improvement relative to another practice or shared benchmark [4]. In the case of pay-for-performance, cross-organization comparisons need to thoroughly address barriers to external validity.

#### Aggregate level

The HDCC had an adequate number of targeted individuals to detect a statistically significant change for each screening test. Yet at the individual health center level, statistically significant changes were observed less than one-half of the time for each cancer screening test, in part due to limited power. The contrast between findings at the individual and aggregate level illustrate one of the strengths of the collaborative model--its potential to demonstrate the collective effectiveness of shared quality improvement efforts that organize individual health centers together. The limitation of combined health center data is the difficulty in specifying where to target interventions based upon aggregate statistics alone. Depending upon stakeholder needs, different methods may be considered for different levels of assessment. Less stringent statistical trends may help to more narrowly target quality improvement resources to the individual health centers struggling in a joint effort. Analytic methods from healthcare systems redesign, such as statistical process control, may be applied to better understand patterns for clinical processes with a small number of observations [34].

Based upon practice registry data, the aggregate collaborative increased screening for breast, cervical, and colorectal cancer. Evidence from other Health Disparities Collaborative programs also suggests positive changes in processes of care [7]. However, twelve months was likely insufficient to distinguish between improvement in clinical performance and improvement in data collection systems. In quality improvement intervention trials, longer follow-up periods are commonly advocated for the sake of better ascertaining sustained improvement [12,35]. In the setting of clinical practices adopting quality improvement goals that track new types of data, longer follow-up periods may also be needed to allow time for the development of new information systems and accompanying workflow processes.

The process of entering patient data into the registry also has potential for selection bias because more active patients (seen in one of the months of collaborative operation) would be more likely to be entered into the registry than patients who had not been seen for some time. The more active patients would also be more likely to have screening and follow up because those were issues covered in the collaborative sessions. There is a reasonable expectation that the relatively inclusive sampling approach to the HDCC's eligible denominator population (seen once in the past three years) underestimates the screening performance, compared to less inclusive sampling approaches to the eligible screening population (for example, if patients were included only if they had been seen in the past year) [36]. Practically speaking, even though the eligible denominator population was standardized and health centers were encouraged to enter that denominator at the beginning of the collaborative, the burden of data entry was considerable, and not all health centers likely could establish the full eligible population by day one of measurement. Thus, centers may have initially been including eligible individuals seen in only the past few months or year. With a less inclusive sampling approach of this type, these centers likely overestimated screening performance. Yet because assessment in the collaborative was primarily done for internal quality improvement, not external reporting purposes, a more inclusive definition of the eligible population was desirable because it can afford centers the opportunity to identify patient populations that might benefit from more intensive outreach [36].

Overall, in this current evaluation of the HDCC program, the validity of the aggregate findings regarding cancer screening is uncertain. Heterogeneous methods of practice registry data collection across a heterogeneous group of health centers (different sizes and approaches to data entry) limit the confidence with which the pooled data can be interpreted and compared to outside organizations. Before external audiences use this type of data as an evaluation tool of an overall collaborative's performance, standardization in the training and experience with the registry is necessary, as well as critical thought about how to consider the various types of heterogeneity across organizations. Again, the HDCC was focused upon quality improvement among participating health centers--not a comparison with other organizations--thus reproducibility and internal validity within participating health centers was the greater priority. Yet, even if internal validity were adequate, our knowledge of temporal trends is limited in a before/after evaluation design with no outside control group. Overall, the findings here do not represent a definitive evaluation of the HDCC. Future collaborative evaluations will benefit greatly from the validation of practice registry data against a 'gold standard', such as paper or electronic medical records, as well as the addition of a control group. Such future evaluations may be expensive, but of course, so are unproven large-scale interventions [37,38].

## Summary

By sharing our unvarnished experience with the HDCC, we have contributed operational knowledge about the implementation and interpretation of practice registries from a quality improvement collaborative. Quality improvement efforts do not routinely perform data validation, although strategic data quality checks would be worthwhile. We have discussed several evaluation design issues, including power, selection bias, and level of analysis. Data collected in the course of quality improvement are commonly imperfect due to their 'real-world' nature; nonetheless, when quantitative measures are used to draw conclusions or support changes in practice, principles of measurement still apply. These principles can provide insight into the limits and potential for the use of practice registry data by stakeholders at both the practice and policy level.

## Competing interests

The authors declare that they have no competing interests.

## Authors' contributions

DH performed the statistical analyses. DH, SC, and ST interpreted the data and drafted the manuscript. ST and DH conceived of the study and participated in its design and execution. All authors read and approved the final manuscript. This work represents the opinion of the authors and cannot be construed to represent the opinion of the U.S. Federal Government.
